# 79. Clinical Utility of Methicillin-Resistant *Staphylococcus aureus* (MRSA) Nasal PCR Assays Beyond Respiratory Infections

**DOI:** 10.1093/ofid/ofab466.281

**Published:** 2021-12-04

**Authors:** Haley M Noeldner, Amanda Bushman, Zach Bliek, Melissa S Wilkinson, Nephi Jones, Ryan Prusa, Rossana M Rosa

**Affiliations:** 1 UnityPoint Health Des Moines - Iowa Methodist Medical Center, Cologne, Minnesota; 2 UnityPoint Health, Des Moines, IA; 3 Iowa Methodist medical center, Waukee, Iowa; 4 Iowa Methodist Medical Center, Des Moines, Iowa

## Abstract

**Background:**

Empiric use of vancomycin is common in clinical practice. Currently there is strong evidence to support the use of MRSA nasal screening to predict the absence of MRSA in respiratory infections; however, minimal data exists regarding its utility as a de-escalation tool beyond pulmonary indications. Furthermore, MRSA nasal PCR has been shown to be a more efficient way to detect the presence of MRSA colonization than traditional culture methods. The purpose of this study was to evaluate the correlation between results of MRSA nasal PCR assays and blood or bone/soft tissue cultures.

**Methods:**

This was a retrospective study of patients who presented to any of three hospitals part of an integrated health system in Des Moines, Iowa, from March 1, 2019 to February 29, 2020. Included patients were those who underwent MRSA nasal PCR screening and had a clinical culture (blood, bone, tissue, deep podiatric wound, joint aspirate, or synovial fluid) obtained within 3 days of the MRSA nasal PCR. Data on age, sex, diabetes mellitus and dialysis were collected. Sensitivity, specificity, positive predictive value (PPV) and negative predictive value (NPV) for all cultures, and blood and bone/soft tissue cultures separately were estimated.

**Results:**

A total of 1989 patients were included in the study. Of these patients, 1953 patients had a blood culture obtained and 171 patients had a bone/soft tissue culture obtained. The median age was 66 years, and 1086 (54.6%) patients were male. At baseline, 33.1% and 3.8% of patients had diabetes or were on dialysis, respectively. The overall prevalence of MRSA colonization was 12.3%. The sensitivities of the MRSA nasal PCR screening were 67.5% for all clinical cultures, 81.8% for blood cultures, and 55% for bone/soft tissue cultures. Specificities were 88.8%, 88.5%, and 92.7% for all cultures, blood cultures, and bone/soft tissue cultures, respectively. The PPVs were 11%, 7.5%, and 50% for all cultures, blood cultures, and bone/soft tissue cultures, respectively, and the NPVs were 99.3%, 99.8%, and 92.7%, respectively.

**Conclusion:**

MRSA nasal PCR screening showed high NPV across blood and bone/soft tissue cultures. These results indicate the clinical utility of MRSA nasal PCR assays beyond respiratory infections and can further support antimicrobial stewardship activities.

**Disclosures:**

**All Authors**: No reported disclosures

